# Plasma Protein Corona Modulates the Vascular Wall Interaction of Drug Carriers in a Material and Donor Specific Manner

**DOI:** 10.1371/journal.pone.0107408

**Published:** 2014-09-17

**Authors:** Daniel J. Sobczynski, Phapanin Charoenphol, Michael J. Heslinga, Peter J. Onyskiw, Katawut Namdee, Alex J. Thompson, Omolola Eniola-Adefeso

**Affiliations:** 1 Department of Chemical Engineering, University of Michigan, Ann Arbor, Michigan, United States of America; 2 Department of Biomedical Engineering, University of Michigan, Ann Arbor, Michigan, United States of America; University of Sassari, Italy

## Abstract

The nanoscale plasma protein interaction with intravenously injected particulate carrier systems is known to modulate their organ distribution and clearance from the bloodstream. However, the role of this plasma protein interaction in prescribing the adhesion of carriers to the vascular wall remains relatively unknown. Here, we show that the adhesion of vascular-targeted poly(lactide-co-glycolic-acid) (PLGA) spheres to endothelial cells is significantly inhibited in human blood flow, with up to 90% reduction in adhesion observed relative to adhesion in simple buffer flow, depending on the particle size and the magnitude and pattern of blood flow. This reduced PLGA adhesion in blood flow is linked to the adsorption of certain high molecular weight plasma proteins on PLGA and is donor specific, where large reductions in particle adhesion in blood flow (>80% relative to buffer) is seen with ∼60% of unique donor bloods while others exhibit moderate to no reductions. The depletion of high molecular weight immunoglobulins from plasma is shown to successfully restore PLGA vascular wall adhesion. The observed plasma protein effect on PLGA is likely due to material characteristics since the effect is not replicated with polystyrene or silica spheres. These particles effectively adhere to the endothelium at a higher level in blood over buffer flow. Overall, understanding how distinct plasma proteins modulate the vascular wall interaction of vascular-targeted carriers of different material characteristics would allow for the design of highly functional delivery vehicles for the treatment of many serious human diseases.

## Introduction

Injectable vascular-targeted carrier (VTC) systems hold great promise for the effective diagnosis and treatment of many human diseases by non-invasively providing localized delivery of imaging agents or potent therapeutics. However, to date, only a few VTCs have been effectively translated into the clinics [1]. One reason for this low clinical success rate may be the lack of a detailed understanding of particle behavior in the intermediate transport step between entry into the bloodstream and binding to the vessel wall. Until now, the evaluation of VTCs for use in human diseases has mostly focused on the development of novel strategies for targeting, e.g. design of unique peptides [2], and formulations that allow for optimal drug release with the general presumption that all VTCs can successfully marginate (localize and adhere to the vascular wall) in blood flow irrespective of size, shape, and material characteristics. However, in the context of VTCs, blood is not a homogenous fluid. Rather, blood in flow is a dense and anisotropic aqueous suspension of mostly red blood cells (RBCs) in the core of flow while white blood cells (WBCs) and platelets in plasma form an outer ring near the wall. In our recent works, we have shown that RBC dynamics and WBC physical interaction affect the ability of particles to marginate as a function of particle size and shape [3]–[5]. However, to date, little is known about the potential role of plasma protein interactions with VTCs in their vascular wall interaction.

The majority of published literature on plasma protein interaction with targeted drug carriers has focused on opsonization [6]–[10], which leads to particle recognition and clearance from the bloodstream by macrophages. Only recently have a few studies reported that the protein corona on nanoparticles (NPs) can interfere with the ligand-receptor interaction, suggesting such impacts highly depend on the targeted cell or protein [11]–[13]. Conversely, others have demonstrated that specific plasma proteins in the particle corona can be exploited to target specific diseased cells [11], [14], resulting in more efficient cellular internalization compared to “naked” NPs [11]. While these studies undoubtedly provide some useful insight into the range of possible impacts of the corona on carrier targeting, researchers have largely focused on impact on specific targeting ligand type (e.g. transferrin) with little emphasis on carrier material composition. It is known that spheres of different material types with identical size and surface charges can adsorb different types and levels of plasma proteins [15], which can result in distinct cellular interactions. Moreover, these previous analyses of corona effects on targeting have been conducted with simple animal sera or culture media in static assays that may not encompass the complexity of the realistic human blood flow environment in which VTCs must function, i.e. presence of hydrodynamic forces and blood cell interactions. To date, it is not clear what effect the nanoscale coating of plasma proteins onto VTC surfaces may have on their interaction with the vascular wall in the complex environment of human blood flow, which is critical for any intravenously administered VTC system designed for human use. In general, the capture and binding of targeted particles to a reactive surface from flow can occur on the order of one to tens of seconds depending on the kinetics of ligand/receptor interaction and provided there is no steric hindrance to receptor-ligand contact or physical barrier to particle localization to the surface [16]. In several experimental works with simple buffer or human blood flows *in vitro*, polystyrene or silica-based microparticles have been shown to effectively localize and bind to the vascular wall [4], [17]–[20]. However, limited work currently exist in the literature on the flow adhesion to reactive surfaces of poly(lactic-co-glycolic-acid) (PLGA) based-particles that are ubiquitously proposed for use as VTCs [21]–[24], all of which have reported on adhesion only in buffer flow assays [25]–[30]. Here, we investigate the role of the plasma protein corona in the adhesion of poly (lactic-co-glycolic-acid) (PLGA) particles to a vascular wall model from human blood flow via *in vitro* assays. Specifically, we characterized the adhesion of sLe^a^-conjugated PLGA particles in laminar and pulsatile human blood flows to a monolayer of activated endothelial cells (ECs) in a parallel plate flow chamber (PPFC). sLe^a^ is a carbohydrate ligand with favorable binding kinetics to E-selectin, overexpressed by inflamed ECs, in flow. This ligand has also previously been proposed for targeting therapeutics in many diseases [31]–[33].

## Materials and Methods

All research involving human participants was approved by the University of Michigan Review Board (IRB). Informed consent, written, was obtained from the participants.

### 2.1. Particle Fabrication and Characterization

The oil-in-water solvent evaporation technique used to fabricate 5 µm PLGA particles has been previously described in the literature [34], [35]. Briefly, 50∶50 PLGA polymer with acid (carboxyl) end group (Evoniks; Parsippany, NJ) was dissolved in 20 ml of dichloromethane (DCM) (oil phase) at 2 mg/mL and injected into 90 mL of polyvinyl alcohol (PVA)/poly(ethylene-*alt*-maleic anhydride) (PEMA) solutions (aqueous phase) under shear. The emulsion is mixed for 2 hr to allow for evaporation of DCM. Differential centrifugation wash steps were employed to reduce residual PVA on the particle surface and overall polydispersity. Finally, the particle solution was frozen and dried on a lyophilizer for ∼1 day. The dried sample is stored dry at −20°C until use. The 500 nm and 2 µm PLGA spheres used were obtained from Phosphorex, Inc. (Hopkinton, MA). These particles were also fabricated from acid (carboxyl)-terminated PLGA polymer with 50∶50 glycolic: lactic co-monomer ratio. The polymer molecular weight for the in-house polymer was 26 kDa and a molecular weight range of 26–30 kDa is reported for the commercial PLGA particles. The average mean particle diameter (Z-average) and polydispersity indexes (PDI) for PLGA spheres in filtered phosphate buffer saline with calcium and magnesium ions (PBS+/+) are measured via dynamic light scattering (DLS) technique using a Malvern Zetasizer Nano ZSP equipped with a back scattering detector (173 degrees). The data obtained is summarized in Table 1. Commercial polystyrene (PS; Bangs Laboratories, IN) and silica (Corpuscular, NY) particles used as control have carboxyl groups on their surfaces similar to PLGA particles. Particle concentration for all assays was determined via a hemacytometer, which contains a grid with specified area. Specifically, a fixed amount (by weight) of particles were suspended in a fixed volume of PBS. The hemacytometer was then used to count the number of particles in a specific volume of solution to yield particle number per mL of solution.

**Table 1:** The average mean particle diameter (Z-average) and polydispersity indexes (PDI) for PLGA spheres in filtered phosphate buffer saline with calcium and magnesium ions (PBS+/+) are measured via dynamic light scattering (DLS) technique using a Malvern Zetasizer Nano ZSP equipped with a back scattering detector (173 degrees).

Particles	Dispersant	Z-average	PDI 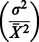
0.49 µm PLGA (YG)	PBS++	334.2 nm	0.232
0.534 µm PS (G)	PBS++	567.0 nm	0.032
2.35 µm PLGA	PBS++	1.407 µm	0.496
2.1 µm PS	PBS++	2.219 µm	0.150
5 µm PLGA	PBS++	4.586 µm	0.243
5 µm PS	PBS++	4.946 µm	0.047

### 2.2. Biomolecule Surface Conjugation

The procedure for covalent conjugation of avidin and the attachment of biotinylated ligands onto carboxylated particles has been previously described by Charoenphol et al. [3], [36]. Briefly, for avidin conjugation, the appropriate amount of polymeric particles equivalent to a fixed surface area of 9.1×10^9^ µm^2^ were suspended per 1 mL of a 5-mg/mL avidin in MES buffer (50 mM) solution for 15 min. Following this step, N-Ethyl-N′-(3-dimethylaminopropyl)carbodiimide hydrochloride (EDAC) was added to the avidin-particle solution to act as a crosslinker such that an EDAC concentration of 37.5 mg/mL and a final avidin concentration of 2.5 mg/mL is achieved. The particle solution pH was adjusted to ∼7.4 and allowed to rotate for 20 hr. A fixed particle surface area is used for conjugation of all particles so as to normalize the avidin coupling condition across the different particle sizes and material types evaluated. The avidin coupling reaction was stopped by addition of 1.5 mg glycine/200 µL of avidin-EDAC-particle solution. Avidin-coated particles were then incubated in a solution of biotinylated sLe^a^ (Glycotech; Gaithersburg, MD) or biotinylated human-anti-ICAM1-mouse-IgG-1 (anti-ICAM-1; R&D Systems; Minneapolis, MN) in PBS +/+ with 1% BSA for 45 min to link targeting ligands to the particle surface. For some assays, 2 µm PLGA particles were first grafted with polyethylene glycol (PEG) chains prior to ligand conjugation to the end of the PEG chains as previously described [20]. Briefly, amine-PEG (5 kDa)-biotin was covalently coupled to the PLGA particles as described for avidin conjugation above. For ligand conjugation onto PEG chains, biotinylated sLe^a^ was premixed with 20 µg/mL NeutrAvidin at an equal volume ratio for 20 min followed by incubation with PEGylated spheres.

### 2.3. Quantification of Particle Surface Ligand Density

Flow cytometry was used to determine the site density of sLe^a,^ and PEG where relevant, on the particle surface as previously described [3], [36]. For sLe^a^ density, targeted particles were thoroughly washed in PBS+/+ and then reconstituted to 1 mL total volume in buffer. A total amount of 5×10^5^ particles for 5µm spheres or 1 and 1.5×10^6^ particles for 2 µm and 500 nm spheres, respectively, were then incubated with a 10 µg/mL solution of anti-CLA-PE (Miltenyi Biotech, San Diego, CA) antibody for 20 min. sLe^a^ coupled particles incubated with rat-IgM isotype was used as control along with sLe^a^ particles without antibody incubation. Stained samples were then thoroughly washed and resuspended in 1 mL of buffer. Flow cytometry was used to measure the fluorescence of the stained (anti-CLA-PE incubated samples) and this shift value was subtracted from the blank sample or isotype control [3], [36]. PE Calibration beads were then used to correlate fluorescence with the number of fluorescent molecules to achieve ligand site density. A similar procedure was used for determination of anti-ICAM-1 and PEG density via goat-anti-mouse IgG-FITC (Jackson ImmunoResearch; West Grove, PA) and avidin-FITC (Thermo Scientific; Waltham, MA), respectively.

### 2.4. HUVEC Culture

Human umbilical vein endothelial cells (HUVEC) were obtained from fresh umbilical cords via a well-known collagenase perfusion method [37]. Human umbilical cords were obtained from the U of M hospital under a Medical School Internal Review Board (IRB-MED) approved human tissue transfer protocol (HUM00026898). This protocol is exempt from informed consent per federal exemption category #4 of the 45 CFR 46.101.(b). Isolated HUVEC were then pooled and grown in tissue culture flasks pretreated with 0.2% w/v gelatin. The growth medium contained 10% fetal bovine serum, 10% bovine calf serum, 1% penicillin-streptomycin, 1% fungizone, 1% HEPES buffer, 1 µg/mL heparin, 50 µg/mL endothelial cell grow supplement, and Medium 199. For each flow channel experiment, HUVEC was cultured onto a 30 mm glass coverslip pretreated with 1% w/v gelatin and cross-linked with 0.5% glutaraldehyde. Coverslips were kept at 37 °C and 5% CO_2_ until confluency of the monolayer was achieved. To activate HUVEC monolayer to promote high expression of E-selectin, IL1-β was added to the cells at a concentration of 1 ng/mL for 4 hours before use [37]. For maximum expression of ICAM-1, coverslips were activated with the IL1-β for 24 hours. The phenotype and expression pattern of primary HUVECs cultured in a monolayer has been fully described by others [38]–[40]. Each activated EC monolayer was used for only one flow assay.

### 2.5. Blood Preparation

Blood was obtained from healthy human donors selected at random via a 60 mL syringe containing citrate anticoagulant (acetate-citrate- dextrose, ACD) according to a protocol approved via the University of Michigan IRB and in line with standards set by the Helsinki Declaration. To obtain pure plasma for incubation, whole blood was centrifuged for 20 min at 2250 g to pellet platelets, white blood cells (WBCs), and red blood cells (RBCs). To isolate RBCs for RBC-in-buffer trials, 1.4 mL of dextran was added per 10 mL of whole blood and after 2 hours was allowed for separation, RBCs were collected and washed, via centrifugation, with PBS without ions.

### 2.6. Parallel Plate Flow Chamber (PPFC) Setup

Flow adhesion assays are performed by use of a circular parallel plate flow chamber with a straight rectangular channel. An activated confluent monolayer of human umbilical vein endothelial cells (HUVEC) was cultured onto a circular class coverslip and attached to a silicon rubber gasket that was vacuum-sealed to the flow chamber deck. Particles coated with either sLe^a^ or anti-ICAM were suspended in blood, plasma, or buffer at a fixed concentration of 5×10^5^/mL for the 2 and 5 µm or 1×10^6^/mL for 500 nm spheres and immediately introduced into the flow chamber via a programmable syringe pump. The flow channel was rinsed with buffer between coverslips and for each trial, individual images of particles bound on cells were taken for ∼10 field of view along the width of the chamber at fixed position from the channel entrance to account for variation in fluxes along the channel length using a Nikon TE 2000-S inverted microscope fitted with a digital camera. All experiments are conducted at 37 °C for 5 min of laminar or 15 min for pulsatile flow. Pulsatile flow profile was generated via a programmable syringe pump and set to pulse around 0 dynes/cm^2^ with net flow in the forward direction at a maximum forward wall shear rate (WSR) of 1000 s^−1^ as previously described [36]. The wall shear rate in the flow chamber for the different trials is calculated by the following equation: 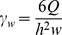


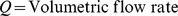


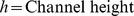


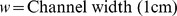


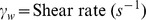



For some assays, PLGA particles were suspended in buffer with viscosity matching that of human plasma (viscous buffer). To achieve this, a 50 Cannon-Fenske viscometer was used to first measure the viscosity of fresh human plasma at 37°C – a value of ∼1.3 cP was achieved. PBS+/+ buffer was then modified with different concentrations of dextran polymer (MW  = 60,000–90,000 Da) and viscosity measured at 37°C similar to plasma. A linear plot of buffer viscosity versus dextran concentration in buffer was generated (data not shown) and used to identify a dextran concentration of 1.4 wt% in PBS+/+ to yield a viscosity that matched that measured for plasma.

### 2.7. SDS-PAGE Gel electrophoresis preparation

Particles (5 µm) for gel electrophoresis were conjugated with avidin and sLe^a^ as described in the biomolecule surface conjugation and quantification section. Following the conjugation steps, particles were incubated in plasma at a concentration of 1.97×10^6^ particles/mL plasma for 1 hr. Plasma incubated particles were then washed 3 times with 50 mM sodium phosphate buffer and heated at 95 C for 5 min in 50 µL of 1× sample buffer (0.3 M Tris- HCl, 5% SDS, 50% glycerol, lane marker tracking dye, pH 6.8) obtained from Pierce SDS-PAGE sample prep kit. Reducing agents such as DTT are optional, and were not added for this assay. SDS gel electrophoresis was performed using Mini-PROTEAN precast gels from BIO-RAD. 10 µL was injected per lane for all sample lanes and 5 µL was used for the molecular weight standard (Precision Plus Dual Color Protein Standards). Run time was 30 min at 200 [V]. A particle (polystyrene or PLGA) surface area of ∼1×10^9^ µm^2^ was used for each sample lane.

### 2.8. Mass spectrometry

Selected gel bands were digested using trypsin and analyzed using LC/MS/MS on a ThermoFisher Velos Orbitrap mass spectrometer. The NCBI protein database allowed for identification of product ion data using the Mascot and X-Tandem Search engines. Output files from Mascot were parsed into Scaffold software to confirm the correct identification of proteins.

### 2.9. Albumin/Immunoglobulin Depletion Kit Assay

Human plasma was diluted 1∶4 using 1× PBS obtained from the PureProteome Albumin-Immunoglobulin depletion kit available from EMD Millipore according to manufacturer recommendation. 100 µL of diluted plasma was added to 900 µL of the PureProteome magnetic beads and incubated for 60 min at room temperature on an end-to-end rotator. The magnetic beads remove >98% of albumin and all immunoglobulin classes (IgG, IgA, IgM, IgD, IgE). Following this incubation step, plasma/bead solutions were centrifuged and the supernatant (depleted plasma) was collected and combined to obtain a total volume of 1 mL. This 1 mL of depleted plasma was then heated to 37 °C and incubated with 3.23×10^6^ PLGA particles for 1 hour and then used in buffer flow adhesion assay via the PPFC. Assays with particle adhesion in albumin-immunoglobulin-depleted plasma flow were not feasible due to the high volume plasma required and the high cost of the kit to deplete 1 mL of plasma.

### 2.10. Data Analysis

Data in figures is plotted with standard error bars and differences in adhesion density was analyzed using one-way ANOVA with Tukey post-test with α = 0.01 (Prism, GraphPad). Adjusted P values of <0.01 were considered significant. Power calculations were done with DSS Research online statistical power calculators.

## Results

### 3.1. Evaluation of PLGA adhesion in human blood flows

**Figure 1:**
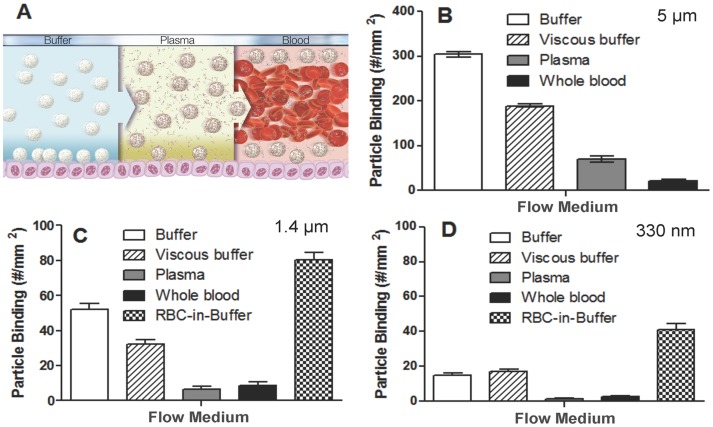
Summary of the adhesion of PLGA spheres from flow of buffer, plasma and blood to an activated endothelial cell monolayer. (A) A depiction of particle margination in buffer, plasma and blood flow. Binding density after 5 min of flow for (B) 5 µm, (C) 1.4 µm and (D) 300 nm sLe^a^-coated pLGA particles to activated HUVEC monolayer from laminar buffer, plasma, or whole blood at 200 s^−1^. Particle concentration  = 5e5 particles/mL for 5 and 1.4 µm data and 1e6 particles/mL for the 330 nm particles. sLe^a^ density  = 1,700+/−100 sites/µm^2^ (SEM) surface for 5 µm, 1500+/−100 sites/µm^2^ (SEM) for 1.4 µm and 9,000+/−400 sites/µm^2^ (SEM) for 330 nm particles. N = 3 distinct donors (donors A, B and C).

The objective of this study is to elucidate the potential role of the human plasma protein coating on drug carrier surfaces in the targeted adhesion of these carriers to the vascular wall under human blood flow conditions (Fig. 1A). PLGA spheres of sizes 5 µm, 2 µm and 500 nm were targeted for use. Based on DLS measurements, the actual average sizes for the 500 nm, 2 µm and 5 µm target size were ∼330 nm, 1.4 µm and 4.6 µm respectively. First, we evaluated the adhesion of PLGA spheres conjugated with a high density of sLe^a^ (>1000 sites/µm^2^) to an activated EC monolayer in low shear (200 s^−1^) laminar flow. On average, the adhesion of the 5 µm PLGA microspheres to activated ECs is 93% lower in human whole blood flow relative to adhesion of the same particles in a simple buffer flow system (Fig. 1B). This discrepancy in particle adhesion between simple buffer versus blood flow is not solely explained by the viscosity difference between the two different mediums since the adhesion level observed for the spheres in blood is still 89% lower than their adhesion in a high viscosity (matching plasma viscosity) buffer flow. Similarly, the EC adhesion of 1.4 µm sLe^a^-conjugated PLGA spheres in low shear laminar blood flow is 83% lower relative to the adhesion of the same particles in simple buffer flow (Fig. 1C). Again, the adhesion level of the 1.4 µm spheres in blood is still 73% lower than their adhesion level in high viscosity buffer flow assays. For the 330 nm sLe^a^-targeted spheres, particle adhesion in the low shear laminar blood flow is 83% less than the observed adhesion in simple buffer, while this same blood adhesion is 85% less than the adhesion of nanospheres in high viscosity buffer (Fig. 1D).

To determine whether the reduced PLGA adhesion in human blood flow is due to particle-blood cell interactions or particle-plasma protein interactions, the EC adhesion of sLe^a^-PLGA spheres was evaluated in plasma (cell-free) flows. Overall, PLGA spheres also exhibit significantly lower adhesion to activated ECs in plasma flows relative to adhesion in viscous buffer flows, with ∼92%, ∼80% and ∼63% reduction in adhesion in plasma flow for the 330 nm, 1.4 µm and 5 µm spheres, respectively. To further confirm the role of plasma proteins, experiments were conducted with particles in laminar flow of human RBCs washed and then suspended in buffer (RBC-in-buffer). The adhesion levels of the 330 nm and 1.4 µm spheres in RBC-in-buffer flow are ∼176% and 55% higher than the values observed in simple buffer flow, respectively. As such, the levels of reduction in the adhesion of these particles in whole blood (essentially RBC-in-plasma) are 94% and 89% lower, respectively, compare to the observed adhesion in their respective RBC-in-buffer assays.

**Figure 2:**
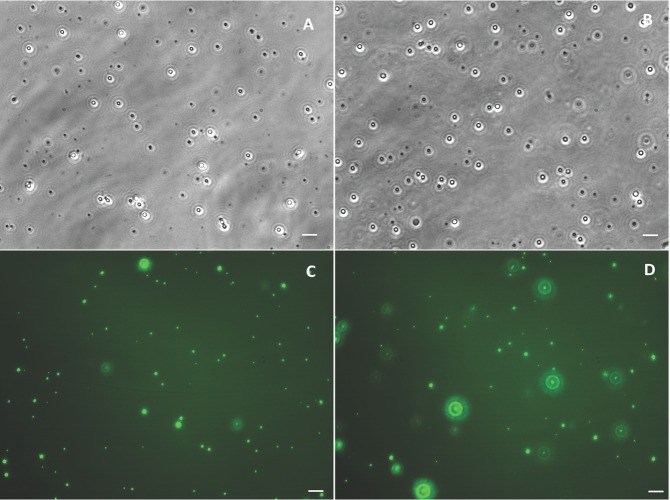
Phase and fluorescence images of small PLGA spheres. Phase image of 1.4 µm PLGA particles in PBS+/+ (A) and plasma (B), and fluorescence image of 330 nm PLGA in PBS+/+ (C) and plasma (D). Particles were added to the desired medium for 5 min in static after which a small amount of the particle solution is placed on a coverslip for imaging. All images shown were taken at a 40× magnification. Scale bar  = 10 µm.

**Figure 3:**
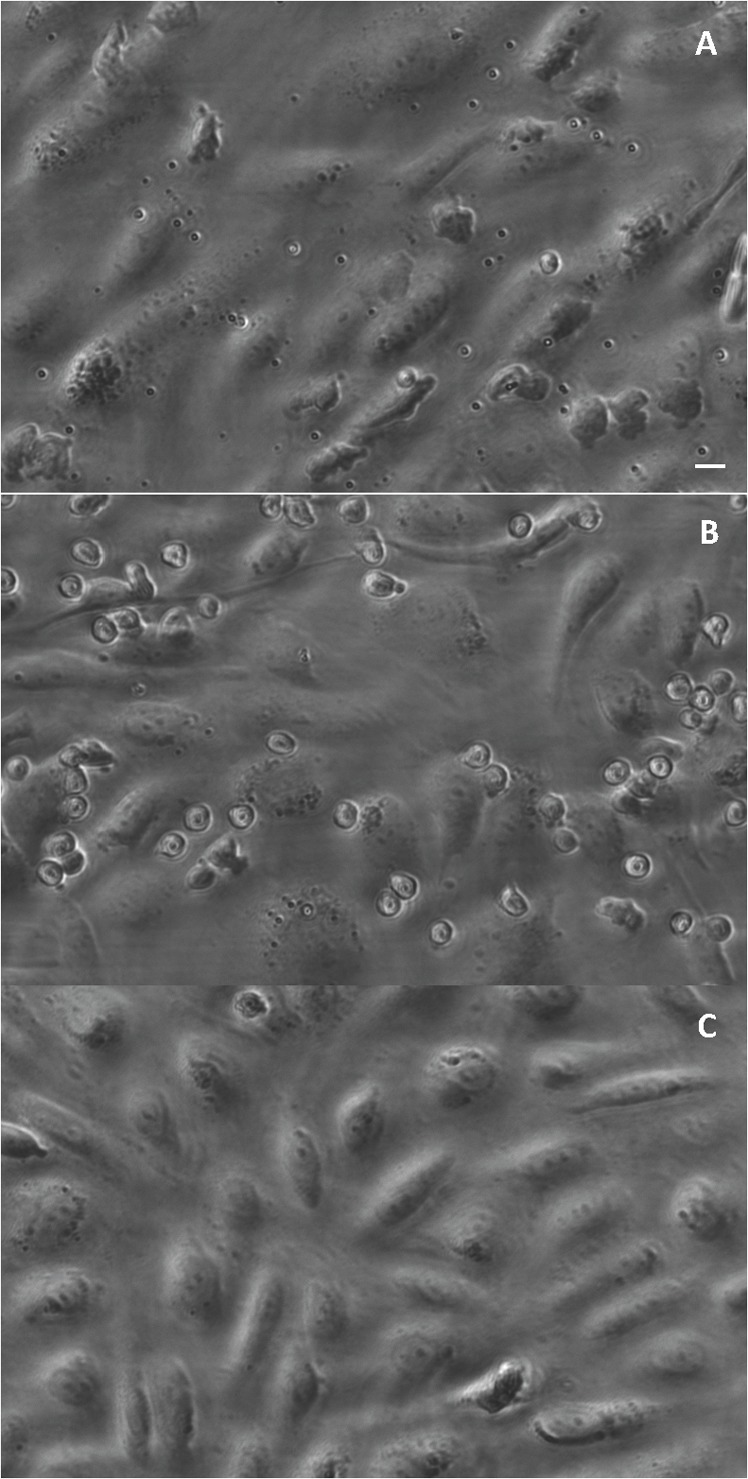
Sample images of activated HUVEC monolayers exposed to PLGA in different flow mediums. Phase image of 1.4 µm sLe^a^-coated PLGA spheres bound to IL1-β-activated HUVEC monolayer after 5 min of flow of particles in (A) RBC-in-Buffer, (B) whole blood, and (C) plasma at 200 s^−1^. Image taken at 20× magnification. sLe^a^ density  = 1500+/−100 sites/µm^2^ (SEM). Particle concentration  = 5e5 particles/mL. Scale bar  = 20 µm

Visualization of particle stability (spheres in solution placed on a glass coverslip) via phase or fluorescent imaging shows all particles to exhibit good dispersity in buffer and plasma (Fig. 2), suggesting that particle aggregation that has been previously reported for some particles, e.g. iron oxide nanoparticles, upon exposure to cell culture media or plasma [41]–[43] is likely not responsible for the observed reduced adhesion of PLGA spheres to ECs in the blood/plasma flow environment. In addition, sample images of the HUVEC monolayer in the PPFC after perfusion of PLGA spheres in RBCs-in-buffer, whole blood and plasma flow are shown in Fig. 3. The bound PLGA particles are easily visualized on the monolayer for the assays with the particles in RBC-in-buffer (Fig. 3A) whereas minimal particles are found on the monolayers perfused with particles in blood (Fig. 3B) or plasma (Fig. 3C). The HUVEC monolayers exposed to RBC-in-buffer and whole blood flows were visually similar and the monolayer integrity is seen to be intact with binding of WBCs visible in both images. The image of the monolayer exposed to particles in cell-free plasma also show essentially no particle binding despite no competition from WBC binding, and a robust cell monolayer remain even after exposure to human plasma.

### 3.2. Evaluation of variation in PLGA adhesion in human blood flows across different donor bloods

**Figure 4:**
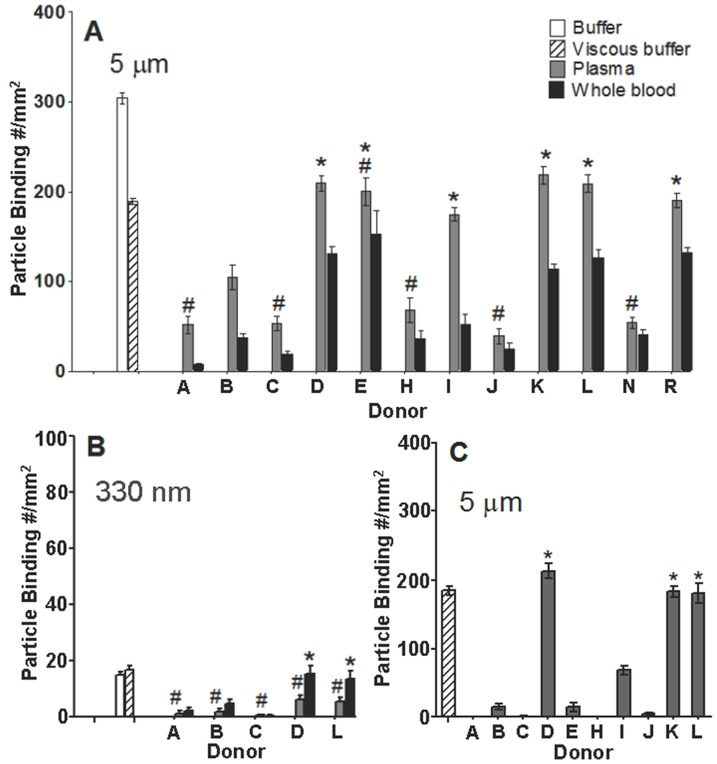
Particle adhesion to activated HUVEC in laminar flow of individual donor plasma and blood at 200 s^−1^. Adhesion of (A) 5 µm and (B) 330 nm PLGA spheres at high sLe^a^ density and (C) 5 µm spheres at low sLe^a^ density. Buffer and viscous buffer controls are shown on the left side of the graph. sLe^a^ density  = 1,800+/−100 sites/µm^2^ (SEM) in (A), 9,000+/−300 sites/µm^2^ (SEM) in (B) and 130+/−10 sites/µm^2^ (SEM) in (C). Adhesion data collected after 5 min of flow time. Particle concentration  = 5e5 particles/mL for 5 µm data and 1e6 particles/mL for the 330 nm particles. #  =  Not significant at 99% confidence relative to whole blood trial from the same donor. *  =  Not significant at 99% confidence relative to viscous buffer. N = 2 distinct assays for each donor.

The adhesion of 5 µm sLe^a^-PLGA spheres in low shear laminar flows of plasma was analyzed for several individual donors to determine any variation in particle adhesion that may be linked to differences in the plasma protein composition of each donor. The 5 µm PLGA sphere size is highlighted in individual donor assays since the magnitude of the reduction in particle adhesion in plasma flow relative to buffer flow is smaller for this particle size compared to the smaller-sized ones. As shown in Fig. 4A, the 5 µm PLGA spheres in flow of plasma from ∼42% of the donors evaluated show an average of 72±15% (SEM) reduction in adhesion relative to adhesion in the viscous buffer control (donors A, C, H, J, N; referred to as “low binding donors” hereafter), whereas there was no evidence of reduced adhesion for the 5 µm PLGA spheres in plasma flow assays with another 50% of donors (donors D, E, I, K, L, R; referred to as “high binding donors” hereafter). Specifically, PLGA adhesion in plasma flow of this subset of high binding donors occurred at a similar level to adhesion in viscous buffer flow. PLGA adhesion in the plasma flow of one donor, donor B, displays an intermediate level of reduction (∼45%) relative to adhesion in viscous buffer. Interestingly, the PLGA adhesion levels between plasma and whole blood assays for the low binding donor bloods are not statistically significant, whereas the opposite is the case for the adhesion of the spheres in the whole blood versus plasma flow assays for most of the high binding donor bloods. To determine whether the donor effect observed for the 5 µm PLGA spheres also exists for smaller spheres, we evaluated the flow adhesion of 330 nm spheres in a subset of the donors evaluated for the 5 µm spheres. Interestingly, while there is minimal impact of plasma proteins seen in the adhesion of the 5 µm PLGA particles in the plasma flow assays with high binding donors compare to viscous buffer, the plasma adhesion of the PLGA nanospheres is statistically lower in both the low and high binding donors than their adhesion in viscous buffer flow (Fig. 4B). However, nanospheres do exhibit a smaller reduction in their adhesion levels in assays with high binding donor plasmas relative to buffer versus the reduction levels observed in assays with low binding donor plasmas (Donors D, L versus A, C). The adhesion of the nanospheres in whole blood flow of the high donors is not statistically different from adhesion in viscous buffer. We also evaluated the individual donor plasma flow adhesion of 5 µm PLGA spheres that were prepared with a low sLe^a^ density (130±10 sites/µm^2^), which is anticipated to increase the available space for a larger amount of proteins to adsorb on particles. The low sLe^a^ particles exhibit significantly low adhesion in plasma flow of 70% of donors relative to adhesion in viscous buffer flow (Fig. 4C) compared to the plasma flow assays with high sLe^a^ density particles where only 42% of donor bloods resulted in a comparable level of reduction in PLGA adhesion relative to viscous buffer.

### 3.3. Evaluation of the effect of flow magnitude and profile on plasma protein effects on PLGA particles

**Figure 5:**
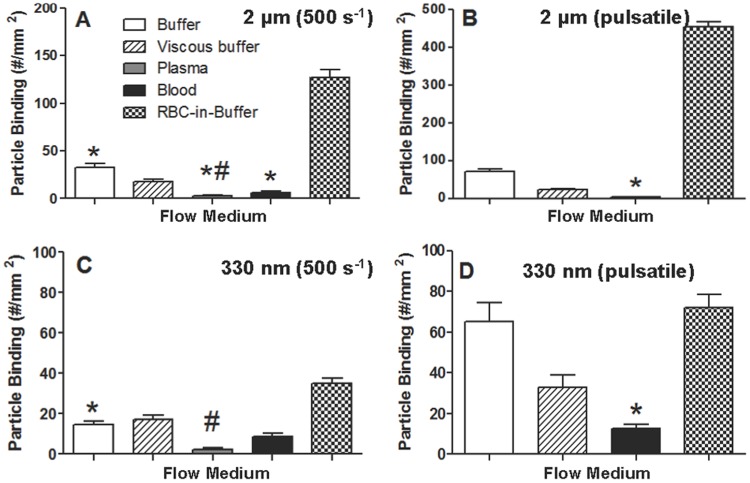
Average adhesion of 1.4 µm and 330 nm sLe^a^–coated PLGA spheres to activated HUVEC in buffer, viscous buffer, plasma, blood and RBC-in-buffer flows. Panels (A) and (B) shows the adhesion of 1.4 µm spheres in laminar shear at 500 s^−1^ and pulsatile flow with peak shear at 1000 s^−1^, respectively. Panels (C) and (D) show the adhesion of 330 nm PLGA spheres in laminar shear at 500 s^−1^ and pulsatile flow with peak shear at 1000 s^−1^, respectively. sLe^a^ density  = 1700+/−100 sites/µm^2^ (SEM) for 1.4 µm spheres and 7000+/−300 sites/µm^2^ (SEM) for 330 nm spheres. Particle concentration  = 5e5 particles/mL for 1.4 µm data and 1e6 particles/mL for the 330 nm particles. Laminar flow was run for 5 min and pulsatile flow for 15 min. #  =  Not significant at 99% confidence relative to whole blood trial from the same donor. *  =  Not significant at 99% confidence relative to viscous buffer. N = 3.

Adhesion of 330 nm and 1.4 µm PLGA spheres were also evaluated in assays with a high shear (500 s^−1^) laminar flow and in a pulsatile flow with alternating forward/reverse flow at high shear (1000 s^−1^) and a net forward flow [44]. As shown in Fig. 5A, the levels of reduction in the adhesion of 1.4 µm spheres in high shear laminar plasma and whole blood flows relative to high shear laminar buffer flow (∼80% and ∼90%, respectively) is similar to the level of reduction in adhesion observed in plasma and whole blood flows versus buffer flow for the low shear laminar assays. However, when adhesion in high shear laminar whole blood flow is compared to that in high shear laminar RBC-in-buffer flow, a slightly higher reduction in adhesion is observed for the 1.4 µm spheres at high shear compared to low shear. Specifically, there is a 95% reduction in blood flow adhesion relative to adhesion in RBC-in-buffer flow at high shear compared to the aforementioned 89% reduction for the same comparison at low shear. In pulsatile flow, the overall adhesion of the 1.4 µm PLGA spheres in blood flow is minimal, with a 95 and 99% reduction in adhesion observed relative to adhesion in buffer and RBC-in-buffer flows, respectively (Fig. 5B). For the 330 nm spheres, the reductions in particle binding in whole blood flow at higher laminar shear and in pulsatile flow relative to simple buffer flows of the same flow type are generally lower than observed for the low shear laminar flow, with 40% and 80% reduction observed, respectively (Fig. 5C–D).

### 3.4. Evaluation of potential plasma protein effects on the flow adhesion of spheres of other material types

**Figure 6:**
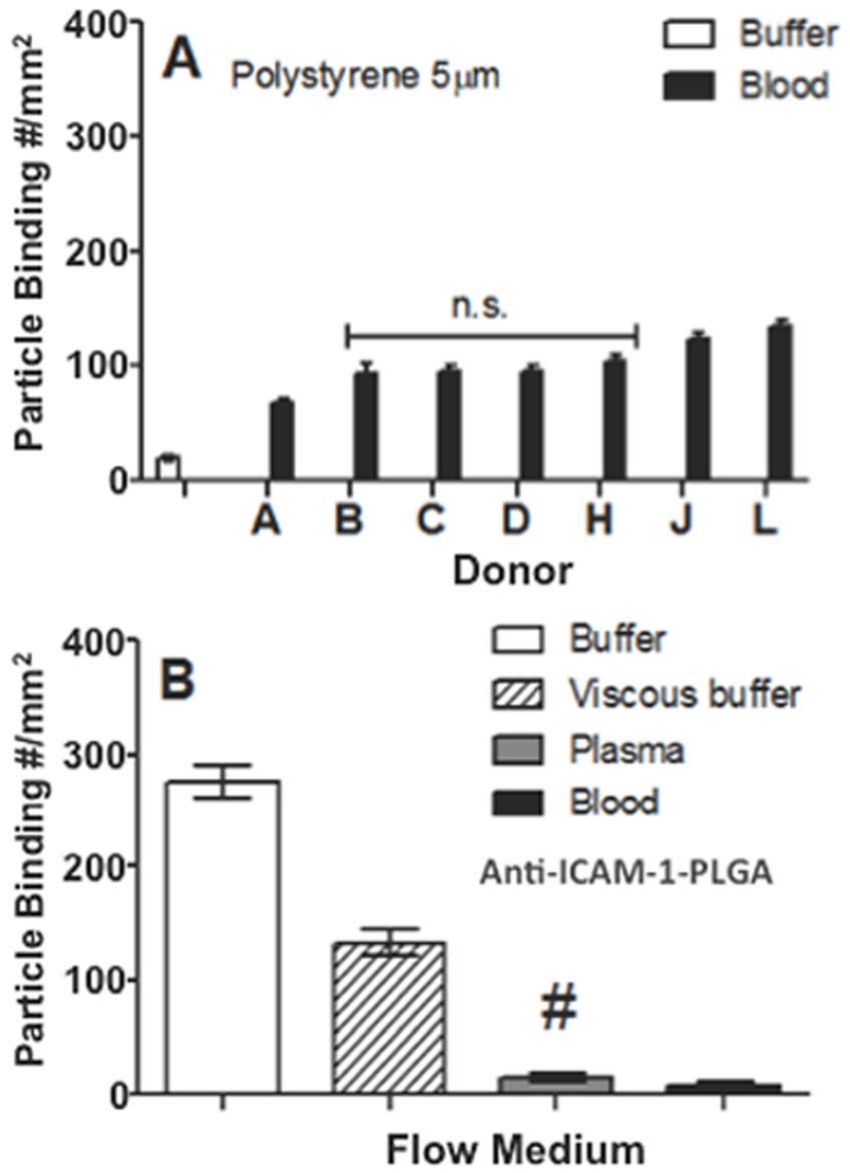
Adhesion of sLe^a^–coated PS spheres or anti-ICAM-coated PLGA spheres to activated HUVEC under various flow conditions. (A) Adhesion of 5 µm sLe^a^–coated PS spheres in laminar whole blood and buffer flows to activated HUVEC at 200 s^−1^ for 7 human subjects. N = 2 (distinct trials) for each blood bar. (B) Average adhesion of 5 µm anti-ICAM-coated PLGA spheres to activated HUVEC from laminar buffer, plasma, or whole blood flow of three low PLGA binding donors at 200 s^−1^. Laminar flow was run for 5 min. Particle concentration in flow  = 5e5 spheres/mL. sLe^a^ density  = 1,800+/−200 sites/µm^2^ (SEM) and anti-ICAM-1 density  = 3500+/−500 sites/µm^2^ (SEM). #  =  Not significant with respect to the whole blood trial. N = 3 distinct trials (donors) for the plasma and blood flow assays.

**Figure 7:**
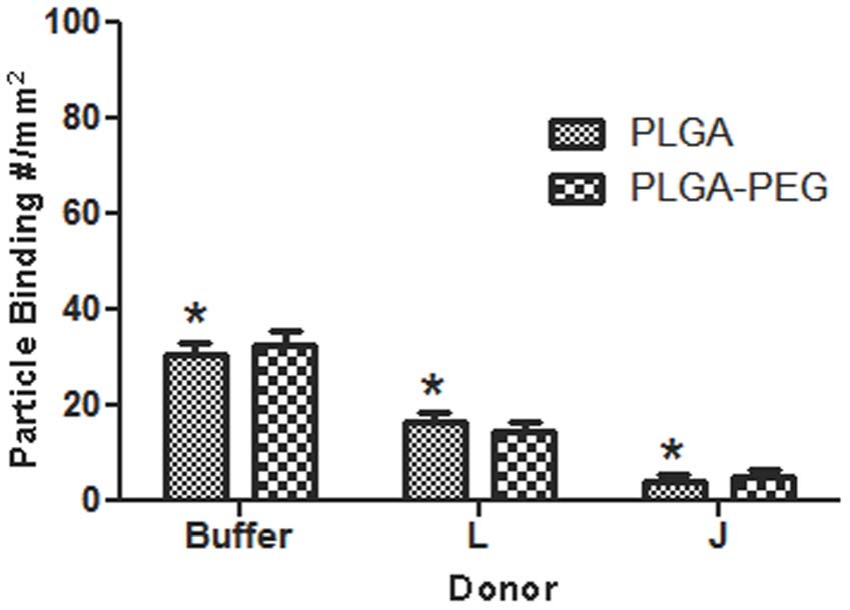
Adhesion of PEGylated and non-PEGylated 1.4 µm sLe^a^-targeted spheres to HUVEC in laminar buffer or whole blood flow at 200 s^−1^ (5 min). A PEG density of 16,000 site/µm^2^ estimated to be the brush conformation is used. sLe^a^ density  = 1,800+/−200 sites/µm^2^ for both PEGylated and un-PEGylated particles. Particle concentration in flow  = 5e5 spheres/mL.

To evaluate whether the observed negative plasma protein effect on particle flow adhesion is unique to PLGA or extends to particles of other material types, we evaluated the adhesion of sLe^a^-coated (high density) polystyrene (PS) spheres to activated ECs from laminar blood flow similar to the PLGA assays. The size and surface charge of the sLe^a^-coated PS were comparable to that of PLGA spheres evaluated (Table S1 in File S1). In contrast to PLGA particles, the adhesion of 5 µm PS particles (similar sLe^a^ density as PLGA particles) in whole blood is higher than their adhesion in buffer flow similar to our previous reports (Fig. 6A) [3], [36]. Moreover, the binding levels of PS in whole blood are comparable among all donor bloods evaluated. As such, there is no significant difference in PS binding between the low binding and high binding donor blood assays contrary to observation with PLGA adhesion, e.g., compare donor D to donors C and H in Fig. 6A. A quick analysis of the blood flow adhesion of sLe^a^-coated silica particles relative to buffer flow also yielded an adhesion trend similar to the trend for PS spheres (Fig. S1 in File S1). This observation with particles of other materials suggests that the plasma protein corona effect observed for PLGA spheres is due to polymer material characteristic, rather than targeting ligand chemistry/interaction, inducing differential plasma protein adsorption. Indeed, when experiments were performed with anti-ICAM-1 (antibody)-coated PLGA spheres, a similar negative binding trend was observed for these particles as was seen with the sLe^a^-coated PLGA spheres (Fig. 6B); yet, in our previous work we show substantial binding of anti-ICAM-1 coated PS microspheres in human blood flow assays [20]. Overall, the successful binding of sLe^a^-coated PS spheres in blood shown in Fig. 6A (and anti-ICAM-1 coated PS spheres in the aforementioned previous publication) highlights that the lack of PLGA binding to ECs in blood flows is not due to human blood components negatively affecting the functionality of the EC-expressed protein targeted. Finally, we evaluated whether incorporation of polyethylene glycol (PEG) chains alters the endothelial cell binding of PLGA particles. PEG chains were conjugated to the surface of 1.4 µm PLGA spheres at a density of 16,000 site/µm^2^ estimated to be in the brush conformation as we previously described [20] and sLe^a^ at high density attached to the chain ends. The PEGylated PLGA spheres demonstrate the same plasma-mediated negative adhesion trend in whole blood flow as observed for non-PEGylated PLGA spheres (Fig. 7). The adhesion trend for a high binding donor (L) versus a low binding donor (J) is also the same as observed for non-PEGylated PLGA spheres. In our previous publication, we show PEGylated 1.4 µm PS spheres to effectively bind to activated ECs from human blood flow [20].

### 3.5. Characterization of protein adsorption on PLGA versus PS spheres

**Figure 8:**
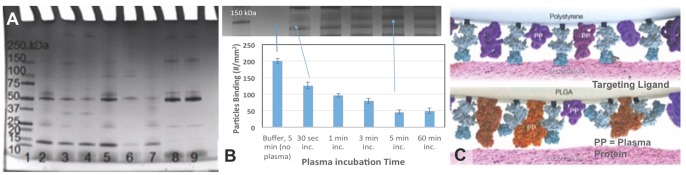
Analysis of proteins adsorbed to particle surfaces as a function of material type and plasma incubation time. (A) SDS-gel electrophoresis analysis of proteins adsorbed on PS (5 µm) and PLGA (5 µm) spheres after 1 hr incubation in plasma. (Lane 1) Molecular weight standard. Proteins adsorbed onto particle surfaces were analyzed from the following conditions: PS (Lane 2) and PLGA (Lane 3) opsonized in a low PLGA binding donor plasma; PLGA (Lane 4) and PS (Lane 5) opsonized in a high PLGA binding donor plasma, and PLGA (Lane 6) and PS (Lane 7) in 1% BSA solution. The protein profile in a 0.4% plasma of the low (Lane 8) and high (Lane 9) PLGA binding donor was also analyzed. (b) Correlation of the buffer flow (200 s^−1^ laminar) adhesion of plasma opsonized PLGA (5 µm) spheres as a function of plasma incubation time (bottom) with the presence/development of a protein band at 150 kDa mark in the protein corona of PLGA spheres as a function of time as observed with SDS-gel electrophoresis analysis (top). (c) A depiction of differential plasma protein (PP) adsorption on PLGA and PS spheres.

To further probe whether the differential protein adsorption on PLGA particles relative to PS is the cause of their differential adhesion to activated ECs in blood flow, we characterized the plasma proteins in the hard corona on sLe^a^-coated particles via SDS-PAGE for all particle sizes evaluated for flow adhesion. Fig. 8A shows a representative SDS gel for the 5 µm PLGA and PS spheres. There were visible differences in the corona acquired by PS microspheres opsonized for 1 hour in plasma (incubation followed by washing in buffer) and the corona found on PLGA spheres (Fig. 8A: Lanes 2 and 5 versus 3 and 4). Interestingly, there were also visible differences in the corona acquired by PLGA opsonized in plasma of a low versus a high PLGA binding donor (Lane 3 versus 4) particularly at the 150 kDa mark where PLGA spheres appear to acquire more proteins in the plasma that supported low PLGA flow adhesion compared to plasma that supported high PLGA binding. The corona acquired by PS microspheres was similar with opsonization in a low or high binding donor (Lane 2 versus 5). Evaluation of the corona of PLGA particles incubated in low binding plasma at various time points, from 0 to 60 min, suggests there is a correlation between the presence and intensity of the 150 kDa band on the SDS gel for PLGA and the extent of reduction in particle blood flow adhesion to ECs as shown in Fig. 8B. The plasma protein interference with binding was also shown to occur as early as 30 sec of particle exposure to plasma/blood and maximizes after only 5 min of exposure. Further analysis via mass spectrometry shows the corona on sLe^a^-PLGA particles incubated with a low PLGA binding plasma contains unique peptides not found in the corona of sLe^a^-PS spheres opsonized in the same donor plasma as depicted in Fig. 8C (Table S2 in File S1). In addition, some protein fragments found in the corona on PLGA in the low PLGA binding plasma were absent in the corona of sLe^a^-PS and sLe^a^-PLGA spheres opsonized with a high PLGA binding plasma (Table S3 in File S1). A majority of unique peptides found on PLGA (particularly when opsonized in low PLGA binding blood) are related to different subclass/subtype of immunoglobulins that belong to a cluster associated with proteins similar in structure to chain L of the insulin growth factor (IgF-Ii) antibody complex.

**Figure 9:**
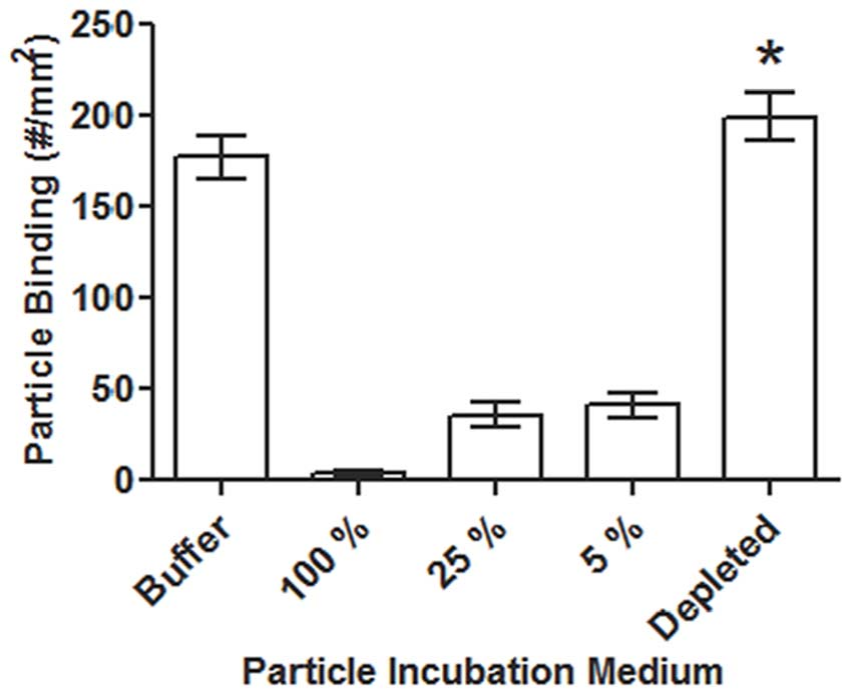
Adhesion of 5 µm PLGA particles at low sLe^a^ density (200+/−200 sites/µm^2^) to HUVEC in laminar buffer flow at 200 s^−1^ (5 min). Particles were soaked for 1 hour in 1 mL of buffer (PBS 1X) or in a low binding donor plasma at 100%, 25%, 5%, or 25% with depletion of albumin/Immunoglobulins prior to flow in buffer for 5 min. *  =  Not significant with respect to buffer trial.

To preliminarily confirm that large immunoglobulins such as ones identified above are involved in the diminished adhesion of PLGA particles in human blood, we evaluated the buffer flow adhesion of sLe^a^-coated PLGA spheres opsonized for 1 hour in 25% plasma of a low PLGA binding donor depleted of 98% of the albumin and all immunoglobulins using a commercial depletion kit (PureProteome Depletion Kit; EMD Millipore) and compared to the adhesion of the same particles soaked in non-depleted plasma of the same donor. In agreement with the SDS-PAGE and mass spectrometry data, PLGA particles opsonized with depleted 25% plasma displayed the same high level of binding in buffer flow as non-opsonized particles whereas particle opsonized in non-depleted, 25% plasma continued to exhibit low adhesion as was the case for non-depleted, 100% plasma (Fig. 9). Furthermore, when the PLGA particles were opsonized with 5% plasma (non-depleted) to account for the reduction in the total protein in the protein-depleted 25% plasma, particle adhesion was still significantly reduced compared to non-opsonized particles.

## Discussion

The data presented here show that vascular-targeted PLGA particles do not effectively adhere to inflamed ECs in human blood flow of different magnitude and flow pattern, an effect that was not observed for PS spheres. We conclude that this phenomena is linked to the unique adsorption of specific “negative proteins” onto the surface of PLGA particles. This conclusion is supported by data from control experiments that shows higher binding of PLGA particles with buffer and RBC-in-buffer flows compare to the values observed in whole blood or plasma only flows of the same flow type and shear magnitude. Also, a preliminary mass spectrometry analysis of the hard protein corona on particles revealed unique proteins, mostly immunoglobulin subclasses/subtypes, found in the protein corona on PLGA but not in the corona on PS particles when exposed to the same donor blood. It is likely that PS do not exhibit reduced adhesion in human blood (or plasma) flow due to the critical negative plasma proteins having a low affinity for these materials. This assertion that different material chemistry affects the type of adsorbed plasma proteins on particles of similar size and surface charge is in line with a previous report by others. Specifically, Deng *et al*. reported that nanospheres of different metal oxides having the same size and surface charge adsorb different plasma proteins [15]. We also find that the negative effect of plasma proteins on the adhesion of PLGA particles in blood flow was persistent for all particle sizes explored, from 5 µm down to 330 nm despite previous report of particle size affecting the quantity and quality of proteins in the corona of nanoparticles [10], [45]. This lack of a major effect of PLGA particle size on their blood flow adhesion to ECs in this work may be due to the affinity of the relevant plasma proteins for PLGA surfaces not being significantly affected within the range of particle size explored. Indeed, about a third or more of plasma proteins in the corona of ultra small nanoparticles are reported to be conserved with changes in particle size [10], [45].

Our results show that the extent of the negative adhesion effect of plasma proteins on PLGA particles is donor dependent, particularly for the largest spherical size evaluated. This “donor effect” is likely linked to variation in plasma protein composition, type and amount of individual proteins, across different individuals, which results in different levels of the “critical” proteins being absorbed on PLGA particles when exposed to different donor blood. Indeed, recent studies have reported the existence of significant plasma protein diversity within the general human population, irrespective of gender and ethnic background [46]. As such, we postulate that PLGA particles in the blood of low binding donors acquire a higher amount of the critical plasma proteins, due to higher abundance of these proteins, which leads to a greater reduction in their EC adhesion in the flow of plasma from these donors than observed in the plasma of high binding donors. This assertion is supported by the observation of a thicker protein band at the 150 kDa protein mark on the SDS-PAGE gel for the corona stripped from 5 µm PLGA particles soaked in plasma from a low binding donor compared to the corona obtained from ones soaked in plasma from a high binding donor. The significantly lower adhesion of the 330 nm spheres in the plasma flow of high binding donors relative to adhesion in viscous buffer versus the lack of a plasma protein impact on the adhesion of 5 µm spheres in the plasma flow of the same high binding donors relative to viscous buffer would suggest that effect of plasma proteins on particle adhesion is more pronounced for the smaller spheres. As aforementioned, protein adsorption processes are the result of thermodynamic gradients that depend on the space available, e.g. particle size, for adsorption to take place [45], [47]. As such, it is likely that the distinction between a high and a low binding donor in terms of plasma protein concentration is less impactful when there is a small surface area for adsorption. However, it is also possible that the larger surface area for contact, and hence more copies of targeting ligand, presented to the EC monolayer by the 5 µm spheres also advantage their binding relative to the smaller spheres for assays conducted at a fixed ligand density (e.g. 1.4 µm spheres), though the larger spheres also experience greater hydrodynamic forces (scale directly with particle diameter [48]) that disrupts their adhesion. Overall, in light of this robust negative effect of plasma corona on the adhesion of the 330 nm PLGA spheres with an actual size range from ∼170–500 nm, we would then anticipate that PLGA nanospheres with sizes in the 50–100 nm range are likely to also exhibit negative adhesion in human blood flow. We are currently working to modify our particle fabrication techniques to obtain PLGA nanoparticles in this size range to confirm this assertion. Finally, though there was no significant donor effect observed with the adhesion of PS spheres in blood relative to buffer flow, the slight reduction in the adhesion of PS particles in the blood of donor A, which consistently conferred the greatest reduction in PLGA adhesion (Fig. 4*A*), compared to adhesion of PS in the blood of other donors may suggest that particles of any material type can have their vascular-targeted adhesion negatively impacted at a high enough plasma concentration of the negative proteins in blood.

The lack of a significance difference in the PLGA adhesion levels between plasma and whole blood flow assays for low PLGA binding donors suggests that the effect of the adsorbed plasma proteins is large enough in these cases, i.e. high adsorption of critical proteins, to make any blood cell-particle interactions that may impact adhesion level inconsequential (Fig. 4*A*: donor A, C, H, J and N). Conversely, the level of adsorption of plasma proteins on PLGA in the blood of high binding donors is likely not as robust such that PLGA adhesion is only mildly affected in the flow of plasma from these donors. However, when particle-blood cell interactions (e.g. collisions) that have previously been reported between microspheres and RBCs and WBCs [3] are present in whole blood flow, it served to further disrupt particle adhesion. This explains the larger reduction in the adhesion of 5 µm spheres in whole blood assays relative to plasma for high binding donors. The distinction between plasma flow adhesion and whole blood adhesion is less pronounced for the smaller spheres evaluated likely due to a reduced (or possibly absent) effect of blood cell-particle interaction for the smaller sizes. We previously reported that the adhesion of 5 µm spheres are significantly reduced in laminar blood flow as the blood hematocrit, or RBC concentration, is increased from 30 to 45% while the adhesion of nanospheres and small microspheres remain the same or is slightly higher with the same increase in blood hematocrit [3]. Here, the presence of RBCs in flow helps concentrate the smaller particles at the wall relative to plasma flow [36] but with no negative impact from blood cell interactions; hence the higher adhesion of the 330 nm particles in whole blood relative to plasma flows for high binding donors. However, the impact of a higher concentration of the 5 µm spheres at the wall in blood flow on their adhesion would be negated by the cell-particle collisions that tend to disrupt adhesion for this particle size. This cell-particle phenomenon also in part explains why the 330 nm and 1.4 µm spheres exhibit significantly enhanced binding in RBC-in-buffer relative to buffer flow (Fig. 1 C and D).

The fact that targeting ligand density affects the adhesion of PLGA particles in different donor blood would suggest that the negative plasma proteins adsorbed on PLGA are interfering with particle-EC binding by weakening the affinity of the receptor-ligand interactions as alluded to in the discussion of the observed donor effect in the previous section. Specifically, in certain donor bloods (i.e., low binding donors) where PLGA particles can attract a high amount of the “negative proteins”, the kinetics of the attached ligand is significantly weakened and hence a large reduction in the capacity of the targeted particle to bind in flow is observed irrespective of a high or low ligand density or, as aforementioned, whether or not the vascular wall interaction is occurring in whole blood (the presence of collisions from blood cells) or plasma flow (Fig. 4). In other donor bloods where the concentrations of the negative plasma proteins are likely appropriately lower, PLGA adhesion is minimally affected in plasma flow when the PLGA particles are at a high targeting ligand density. However, there is still some moderate weakening of ligand kinetics occurring in these cases, which is highlighted by the significantly reduced particle adhesion when a low targeting ligand density (donors B, E and I) is present on the particle surface. For these donor bloods, the exaggeration of the negative adhesion of PLGA particles at low ligand density relative to high ligand density may not necessarily be due solely to greater changes in ligand affinity associated with a larger amount of adsorbed proteins on particles but could also be due to low avidity of the moderately weakened ligands. That is, there is not enough of the moderately weakened ligand to support adhesion at a fixed wall shear stress in flow.

Finally, it is not surprising that PEGylation of PLGA particles has no apparent effect on their negative adhesion in human blood. While the use of PEG spacers located between the material surface and the targeting ligand is a strategy that is often employed to reduce protein adsorption on VTCs, it is known that protein adsorption is always present at some basal level on PEG grafted VTCs. For example, Gref et al. showed that PEG-coated surfaces can still support protein adsorption via direct interactions with the core material even at high PEG density, poly(lactic acid) (PLA), PLGA and poly(varepsilon-caprolactone) (PCL) nanoparticles coated with the same high density of PEG where shown to adsorb different levels and types of protein on their surfaces [49]. However, it remains possible that at a ultra-high PEG density on carrier surfaces that results in no protein absorption onto carrier surface the negative effect of plasma proteins on PLGA carriers can be eliminated [50]. In our ongoing work, we are exploring whether variation in PEG density and chain length can alter PLGA particle adhesion to the vascular wall in blood.

## Conclusions

In this work, the adsorption of certain plasma proteins from human blood onto PLGA carriers were found to prevent these particles from effectively adhering to activated endothelial cells in *in vitro* assays, an effect that was not observed for particles of other material types. Our results also show that the extent of the negative adhesion effect of plasma proteins on PLGA particles is dependent on specific blood donors and the targeting ligand density but not the targeting ligand type. Overall, the presented data suggests that specific knowledge of the plasma protein composition across different humans may be critical to VTC design and their successful clinical use, i.e., highlighting the need for a shift toward personalized medicine in the design of targeted therapeutics. Alternatively, it is possible that with a detailed understanding of the specific proteins that affect particle vascular targeting, novel biomaterials can be designed to resist the adsorption of these proteins in order to achieve enhanced vascular targeting irrespective of the plasma composition of different individuals. A potential limitation to this study, however, is in our evaluation of blood flow adhesion *in vitro* over culture endothelial cells. A detailed conclusion of the effect of plasma proteins on particle margination may necessitate evaluation *in vivo* in animal models – though differences in plasma protein composition between human and common animals used in experimental research may complicate such analysis. Our future studies will aim to specifically identify which individual plasma proteins in human blood are associated with low PLGA margination as well as to further investigate the existence of this plasma protein effect for other biomaterials. Furthermore, we will conduct preliminary *in vitro* assays of particle margination in mouse blood flow to identify any potential difference in PLGA (and other biomaterials) margination relative to human blood as a first step toward future *in vivo* analysis of plasma protein modulation of vascular-targeted particle margination.

## Supporting Information

File S1 (DOC)
