# Supplementary material for: Plasma Protein Corona Modulates the Vascular Wall Interaction of Drug Carriers in a Material and Donor Specific Manner
Source: PLoS One. 2014 Sep 17;9(9):e107408. doi: 10.1371/journal.pone.0107408 (PMC4168002; doi:10.1371/journal.pone.0107408)
Supplement: File S1 — (DOC) [file pone.0107408.s001.doc]

Supplementary Materials

**Fig. S1**: Adhesion of 500 nm sLe^a^-coated silica spheres to activated HUVEC from laminar buffer and human red blood cell (RBC) in plasma flow at 500 s^-1^. Particle concentration in flow = 5e5 spheres/mL. sLe^a^ density = ~1000 sites/μm^2^. N = 5

**Table S1.** Sample Zeta potential measurements for 2 *µm* spheres.

**Analysis of protein corona on PLGA and polystyrene particles**

Data was obtained via mass spectrometry. Proteins striped off particle surfaces were digested into peptide fragments and the identified peptides on the different particles are listed below. ExPASy and other search engines can be used to determine the proteins corresponding to the listed peptide fragments. Single digit spectral counts are taken to be trace amounts and is considered a negligible amount of identified peptide. Unique peptides listed were analyzed via Fisher’s exact test, and a P-value < .01 was considered statistically significant. Molecular weight (MW) of the peptide is also listed in kDa. Table S2 lists peptides that were uniquely found in non-trace amounts on poly(lactic-c-glycolic acid) (PLGA) but not polystyrene (PS) particles. Table S3 lists peptides uniquely adsorbed on PLGA particles incubated in a low PLGA binding donor plasma but not on polystyrene particles incubated in the same donor plasma or PLGA particles incubated in a high PLGA binding donor plasma. Table S4 lists peptides uniquely found in non-trace amounts on polystyrene particles but not PLGA particles incubated in the same donor plasma. The cluster of proteins from which the peptide fragments belong to is also listed for Tables S2 and S3.

**Table S2: Unique peptides found on PLGA particles incubated in a low or high PLGA binding human** plasma

| **#** |  | **Molecular Weight (kDa)** |
| --- | --- | --- |
| **Cluster of Chain L, Crystal Structure Of Igf-Ii Antibody Complex** | | |
| 1 | protein Len,Bence-Jones | 24 |
| 2 | recombinant IgG4 heavy chain [Homo sapiens] | 43 |
| 3 | Chain A, Human Factor Viii C2 Domain Complexed To Human Monoclonal Bo2c11 Fab | 23 |
| 4 | immunoglobulin lambda 2 light chain [Homo sapiens] | 23 |
| 5 | Chain H, Crystal Structure Of Human Anti-Steroid Fab 5f2 In Complex With Testosterone | 24 |
| 6 | immunoglobulin variable region [Homo sapiens] | 17 |
| 7 | unnamed protein product [Homo sapiens] | 55 |
| 8 | Chain A, Crystal Structure Of An Autoimmune Complex Between A Human Igm Rheumatoid Factor And Igg1 Fc Reveals A Novel Fc Epitope And Evidence For Affinity Maturation | 26 |
| 9 | Chain L, Crystal Structure Of Human 2909 Fab, A Quaternary Structure-Specific Antibody Against Hiv-1 | 23 |
| 10 | Chain L, Crystal Structure Of Fab Del2d1, A Deletion Variant Of Anti-Influenza Antibody 2d1 | 23 |
| **Cluster of actin, cytoplasmic 2 [Homo sapiens]** | | |
| 11 | POTE ankyrin domain family member F [Homo sapiens] | 121 |
| 12 | PREDICTED: actin, aortic smooth muscle isoform 2 [Equus caballus] | 37 |
| **Cluster of v-src sarcoma (Schmidt-Ruppin A-2) viral oncogene homolog (avian) [Homo sapiens]** | | |
| 13 | Chain A, Crystal Structure Of Src Kinase Domain In Complex With Cgp77675 | 32 |
| **Complement factor H-related protein 5 precursor** | | |
| 14 | complement factor H-related protein 5 precursor [Homo sapiens] | 64 |
| **Cluster of unnamed protein product** | | |
| 15 | unnamed protein product [Homo sapiens] | 141 |
| **Cluster of growth-inhibiting protein 25 [Homo sapiens]** | | |
| 16 | Chain A, Crystal Structure Of Cleaved Human Alpha1-Antichymotrypsin At 2.7 Angstroms Resolution And Its Comparison With Other Serpins | 41 |
| 17 | Chain A, Alpha1-Antichymotrypsin Serpin In The Delta Conformation (Partial Loop Insertion) | 45 |
| **Apolipoprotein A-V precursor [Homo sapiens]** | | |
| 18 | apolipoprotein A-V precursor [Homo sapiens] | 41 |
| **Cluster of unnamed protein product [Homo sapiens]** | | |
| 19 | unnamed protein product [Homo sapiens] | 139 |

**Table S3: Unique peptides found on PLGA particles incubated in a low PLGA binding donor plasma and not on PLGA particles incubated in high binding donor plasma or on Polystyrene particles incubated in plasma**

| **#** |  | **Molecular Weight (kDa)** |
| --- | --- | --- |
| **Cluster of Chain L, Crystal Structure Of Igf-Ii Antibody Complex** | | |
| 1 | immunoglobulin light chain [Homo sapiens] | 23 |
| 2 | Ig lambda chain - human | 25 |
| 3 | immunoglobulin lambda 2 light chain [Homo sapiens] | 23 |
| 4 | immunoglobulin lambda light chain VLJ region [Homo sapiens] | 28 |
| 5 | Ig A L | 23 |
| 6 | Ig lambda chain NIG76 precursor - human | 23 |
| 7 | immunoglobulin light chain variable region [Homo sapiens] | 23 |
| 8 | Ig nonfunctional kappa-chain (C-region), partial [Homo sapiens] | 12 |
| 9 | immunoglobulin light chain [Homo sapiens] | 24 |
| 10 | immunoglobulin lambda light chain, partial [Homo sapiens] | 23 |
| 11 | immunoglobulin lambda 2 light chain [Homo sapiens] | 23 |
| 12 | Chain L, Tr1.9 Fab Fragment Of A Human Igg1 Kappa Autoantibody | 23 |
| 13 | IgM kappa chain [Homo sapiens] | 24 |
| 14 | Chain L, Crystal Structure Of Anti-Hiv-1 Fab 537-10d In Complex With V3 Peptide Mn | 23 |
| 15 | unnamed protein product [Homo sapiens] | 57 |
| 16 | immunoglobulin lambda light chain VLJ region [Homo sapiens] | 28 |
| 17 | Chain L, Crystal Structure Of The Neutralizing Fab Fragment Abd1556 Bound To The Bmp Type I Receptor Ia | 22 |
| 18 | immunoglobulin kappa light chain variable region [Homo sapiens] | 16 |
| **Cluster of histidine-rich glycoprotein precursor [Homo sapiens]** | | |
| 19 | unnamed protein product [Homo sapiens] | 60 |
| **Cluster of beta-1-syntrophin [Homo sapiens]** | | |
| 20 | syntrophin, beta 1 (dystrophin-associated protein A1, 59kDa, basic component 1) [Homo sapiens] | 58 |

**Table S4: Unique peptides found on polystyrene particles incubated in human plasma but not on PLGA incubated in human plasma**

| **#** |  | **Molecular Weight (kDa)** |
| --- | --- | --- |
| 1 | protein Rei,Bence-Jones | 24 |
| 2 | immunoglobulin mu heavy chain, partial [Homo sapiens] | 66 |
| 3 | PREDICTED: LOW QUALITY PROTEIN: POTE ankyrin domain family member J [Homo sapiens] | 118 |
| 4 | albumin, isoform CRA_a [Homo sapiens] | 25 |
| 5 | complement component 5 variant [Homo sapiens] | 123 |
| 6 | unnamed protein product [Homo sapiens] | 58 |
| 7 | unnamed protein product [Homo sapiens] | 52 |
| 8 | Tubulin, beta [Homo sapiens] | 50 |
| 9 | tubulin alpha-8 chain isoform 1 [Homo sapiens] | 50 |
| 10 | plasma serine protease inhibitor preproprotein [Homo sapiens] | 46 |
| 11 | unnamed protein product [Homo sapiens] | 59 |
| 12 | unnamed protein product [Homo sapiens] | 61 |
| 13 | unnamed protein product [Homo sapiens] | 62 |
| 14 | inter-alpha (globulin) inhibitor H1, isoform CRA_b [Homo sapiens] | 99 |
| 15 | Chain A, Crystal Structure Of Human Phosphoglycerate Kinase Bound To D-Adp | 45 |
| 16 | unnamed protein product [Homo sapiens] | 57 |
| 17 | plasma protease (C1) inhibitor precursor [Homo sapiens] | 55 |
| 18 | unnamed protein product [Homo sapiens] | 137 |
| 19 | Chain A, X-Ray Structure Of Bikunin From The Human Inter-Alpha-Inhibitor Complex | 16 |
| 20 | unnamed protein product [Homo sapiens] | 77 |
| 21 | integrin alpha-6 isoform b precursor [Homo sapiens] | 119 |
| 22 | inter-alpha (globulin) inhibitor H4 (plasma Kallikrein-sensitive glycoprotein), isoform CRA_b [Homo sapiens] | 101 |
| 23 | integrin alpha-2 precursor [Homo sapiens] | 129 |
